# A New World Health Era

**DOI:** 10.9745/GHSP-D-17-00297

**Published:** 2018-03-21

**Authors:** Ariel Pablos-Méndez, Mario C Raviglione

**Affiliations:** a Columbia University Medical Center, New York, NY, USA. Formerly the Assistant Administrator for Global Health, United States Agency for International Development, Washington, DC, USA.; b University of Milan, Italy. Formerly Director of the Global Tuberculosis Programme, World Health Organization, Geneva, Switzerland.

## Abstract

Unprecedented economic progress and demands for social protection have engendered an economic transition in health in many low- and middle-income countries, characterized by major increases in domestic health spending and growing national autonomy. At the global level, development assistance is refocusing on fragile states, the poorest communities, and cooperation on global public goods like health security, technical norms, and innovation. Intergovernmental organizations like WHO need the wherewithal and support to provide leadership and to properly advance this new world health era.

Between 2010 and 2015, development assistance for health (DAH) reached over US$30 billion a year,^1^ and the Millennium Development Goals (MDGs)^2^ helped drive unprecedented gains in development and health equity.^3^ While those accomplishments are cause for celebration,^4^ DAH budgets have tightened^1^ as the world confronts new health challenges, and the global health community is worried about human rights reversals by recently elected populist governments.^5^ Health financing at the country level looks more promising and could be the basis for a new world health era.

## AN ECONOMIC TRANSITION IN HEALTH

After centuries of flat incomes per capita, the world has experienced a 20-fold increase in gross domestic product (GDP) during the last 50 years.^6^^,^^7^ The majority of countries that were considered low-income in 1990, including Bolivia, Bangladesh, and the Republic of Congo, have moved to lower-middle or middle-income status.^8^

Health spending is very closely correlated with GDP and it accounts for an expanding fraction of any growing economy.^9^ While that is often a fiscal and political headache for richer countries, for a growing number of lower-income countries the increase in health resources has the potential to cover the average cost per capita of essential lifesaving commodities and services.^9^

Health spending accounts for an expanding fraction of total spending for any growing economy.

**FIGURE 1.**
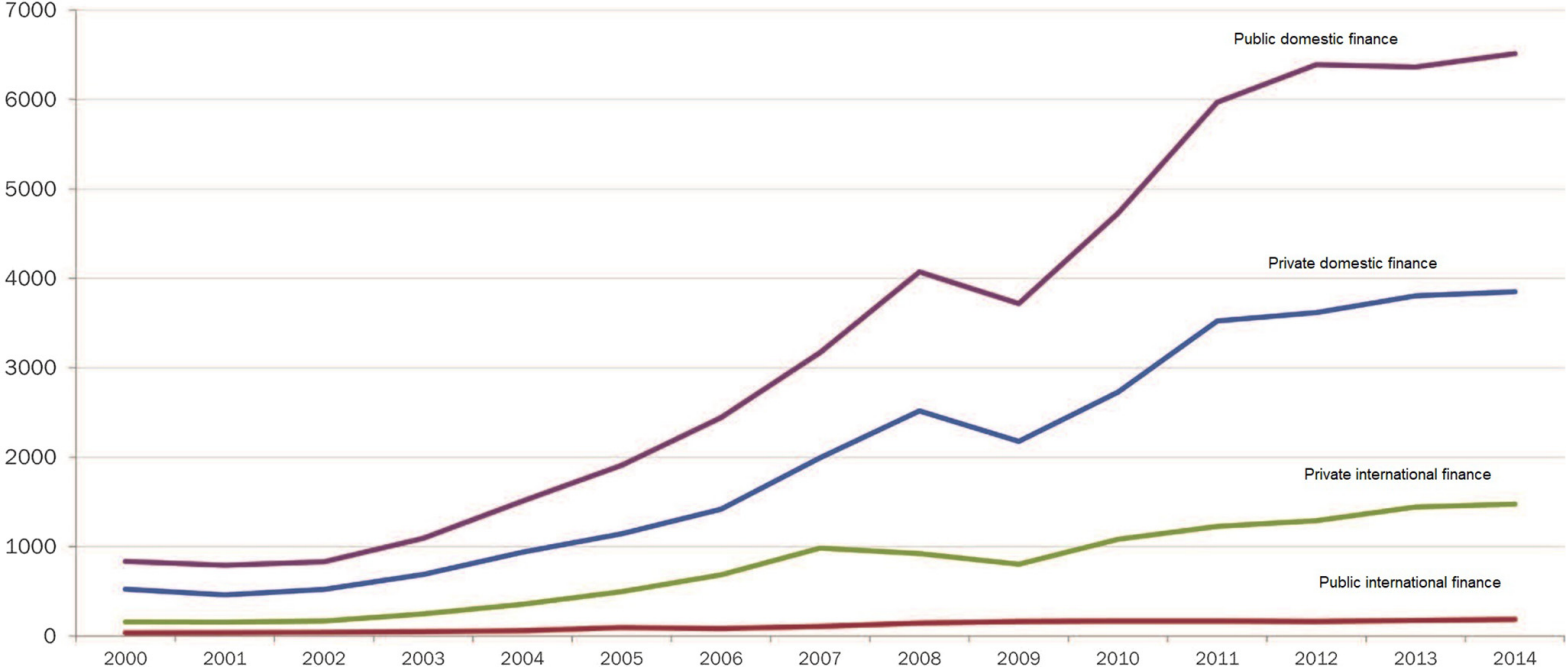
Financing Trends in Developing Countries, 2000–2014 (in US$, billions, 2013 prices) Note: Public domestic finance is defined as total government revenue. Gross-fixed capital formation by the private sector was used as an indicator for private domestic finance. Private international finance is the sum of foreign direct investment, portfolio equity and bonds, commercial banking and other lending, and personal remittances. Public international finance equals the total official flows (official development assistance and other official flows). Source: United Nations Research Institute for Social Development (2016).^10^

As DAH plateaued in recent years,^1^ many low-income countries saw total health spending grow at 10% per year (based on data from National Health Accounts compiled by USAID in 2015). Public and private domestic resources now dwarf DAH (Figure 1). The growth of health spending, however, follows a surge in the demand for health services that is often met by unregulated private services paid out-of-pocket, an inefficient and regressive form of health financing.^11^ This transition is linked to the economics of countries at different stages of development. Thus, these changes have already occurred in several countries and may not be complete in others by 2030.

## A HISTORICAL PENDULUM IN THE POLITICAL ECONOMY

Political economy is the branch of social science that studies the relationships between individuals and society and between markets and the state.^12^ The liberal forces galvanized by the Enlightenment, the 18th century philosophical movement in Europe that promoted freedom, fraternity, solidarity, and equality, have brought unprecedented well-being to our civilization,^13^ but progress has not been linear. Periodic structural shifts in the political economy, whether arising from global crises or national elections, bring new challenges and opportunities and change the ways in which the health agenda is advanced.

**FIGURE 2.**
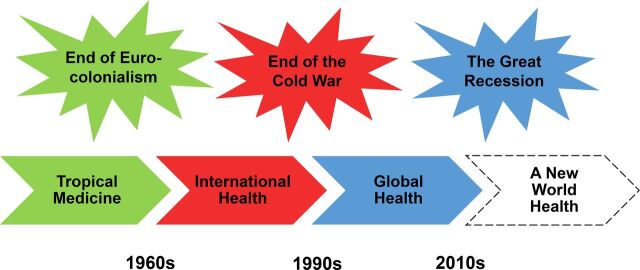
A New Chapter in International Health History Note: This graph is only a didactic tool; historical periods often overlap and vary from one country or region to another, and many components of one period carry over to future ones.

After World War II, with the end of European colonialism, what was known as geographic or “tropical medicine” became firmly established as “international health,” with newly created international agencies and new and assertive nations committed to primary health care (Figure 2).^14^ The World Health Organization (WHO) was the unquestionable leader of the period, but its uniqueness started being challenged in the early 1990s.^15^^–^^18^

At that time, after the collapse of the Soviet Union, the Washington Consensus—the set of market economic ideas supported by the International Monetary Fund and the World Bank—downplayed national government and promoted neoliberal policies, i.e., a strong market-based approach to globalization, decentralization, and privatization.^19^ New philanthropy and the AIDS movement were additional ingredients of what became “global health.”^20^ WHO's influence waned^21^^,^^22^ amidst a plethora of new public-private partnerships advancing donor-supported initiatives against AIDS, tuberculosis, malaria, and vaccine-preventable diseases.^14^^,^^23^ While some experts worried about open-source anarchy and undue influence,^24^ these global partnerships contributed to achieving several MDGs.

We are at a new inflexion point. The Great Recession of 2008 caused a reduction in global GDP and global trade for the first time in half a century.^25^ While markets have recovered since, their failure caused ongoing social pain and revealed severe inequalities,^26^ leading to economic insecurity and growing political demand for social protection and popular rejection of globalization.^5^^,^^27^

National governments are reasserting themselves, in extreme cases with protectionism and xenophobia.^28^ Countries that responded to the crisis with fiscal austerity have faced a wave of antiestablishment, ethnopopulist elections not seen since the 1930s.^29^ This creates many domestic problems of its own and pushes back against the international cooperation and altruism that characterized the golden era of global health. In the long run, populism is no substitute for sound governance and it carries risks.^30^ Good technical and political leadership is needed to address the underlying economic inequalities responsible for the social turmoil seen in many countries.

Good technical and political leadership is needed to address the underlying economic inequalities responsible for the social turmoil seen in many countries.

## A NEW WORLD HEALTH ERA?

Economic growth and increasing health spending in many “developing” countries, along with stagnant DAH and a wave of populist policies, pave the way for a number of profound changes in our field (Table). At the national level, there will be more country ownership and domestic resource mobilization (DRM), with an increasingly feasible possibility of achieving universal health coverage (UHC). At the global level, the power of DAH is diluted and likely to refocus on fragile states and global public goods with benefits to all countries (see below). As a result, member-state organizations like WHO and the World Bank, in coordination with other influential public and private actors, have new opportunities to address existing and emerging health challenges.^31^ Many existing organizations at the global and national level will adjust the role they play and how they fund their work in this new world health era.

**TABLE.** Changes in the Health Field, 1960s to Present Day

Period	International Health 1960s to 1990s	Global Health 1990s to circa 2015	New World Health 2008 to Present
Geopolitical origins	End of European Colonialism with new voting members in a newly formed UN	End of the Cold War (and the Soviet Union), freer trade, Internet, and AIDS	Financial markets crash, OECD recession, and emergent developing economies
Political economy tone	Cold War with East-West divide	“Government is the problem,” markets and civil society the solution	Reassertion of nation-state and demands for social protection
Construction of health	WHO holistic definition and social construction of health	Simultaneously, human rights and reductionist technology	Multisectoral, social determinants, and universalism
Predominant approach	Primary health care, “Health for All,” and solidarity as universal principles and movements	Top-down programs and PPPs to fight key diseases of poverty in developing countries	Grand convergence between North and South, progressive realization of UHC and global health security
International cooperation	Colored by foreign affairs (East-West competition, with exceptions like smallpox eradication)	Explosion of NGOs, PPPs, and new philanthropy tackling the MDGs in poor countries	Assertive but interdependent nation-states sign up to the universal SDGs
Development assistance for health	Newly created UN agencies like WHO and bilateral donors like USAID	Billion-dollar platforms (Gavi, The Global Fund, PEPFAR), Bill & Melinda Gates Foundation a major player, *golden era of DAH*	DAH stagnation, domestic resource mobilization, and graduation from assistance (except fragile states)
Governance	WHO takes center stage in the UN architecture	“Open source anarchy” (WHO's authority diluted)	Sovereign states reasserted; opportunity for WHO
Private sector	Essentially proscribed from UN settings and agenda	Rise in prominence both through PPPs and philanthropy, IT enables global communications	Half of the health sector provision and growing markets in emerging economies
Civil society and community role	Empowerment of communities after Alma-Ata Declaration of 1978	Growing activism, especially linked with HIV/AIDS	National NGOs very important despite closing space in some countries

Abbreviations: DAH, development assistance for health; IT, information technology; MDGs, Millennium Development Goals; OECD, Organisation for Economic Co-operation and Development; PEPFAR, U.S. President's Emergency Plan for AIDS Relief; PPPs, public-private partnerships; SDGs, Sustainable Development Goals; UHC, universal health coverage; UN, United Nations; USAID, United States Agency for International Development; WHO, World Health Organization.

### Domestic Resource Mobilization

With emerging economies growing, the Third International Conference on Financing for Development, held in Addis Ababa, Ethiopia, positioned DRM at the heart of the post-2015 agenda.^32^ The World Bank estimates that simply bringing laggards to the median government revenue level by increasing tax ratios to the median 23% of GDP in low- and middle-income countries would add US$26 billion each year for public expenditure in health.^33^ In addition, increasing the government budget allocation for health to just the median level of 10% would generate an extra $50 billion each year.^33^ Tobacco taxes can contribute to general taxation and also reduce one of the main drivers for chronic diseases. Additional DRM possibilities include leveraging concessionary loans from development banks (e.g. the Global Financing Facility),^34^ innovative financing (e.g., social impact bonds, loan guarantees),^35^ and shaping responsible markets.^36^ Countries like Ghana, Ethiopia, and Rwanda have shown it is possible to increase health budgets significantly.

### Universal Health Coverage

As shown in Figure 1, health spending is rising and will likely continue to do so as GDP expands. In the absence of government policy on public or private insurance, health spending is often paid out-of-pocket by individuals, which sends millions of families back into poverty.^37^^,^^38^ Such expenditures account for 50% of total health spending in most African countries and up to 80% in large South Asian nations—versus less than 20% in most countries of the Organisation for Economic Co-operation and Development (OECD).^11^ In response to this growing challenge, UHC is becoming the organizing principle for health systems everywhere.^39^^,^^40^

**FIGURE 3.**
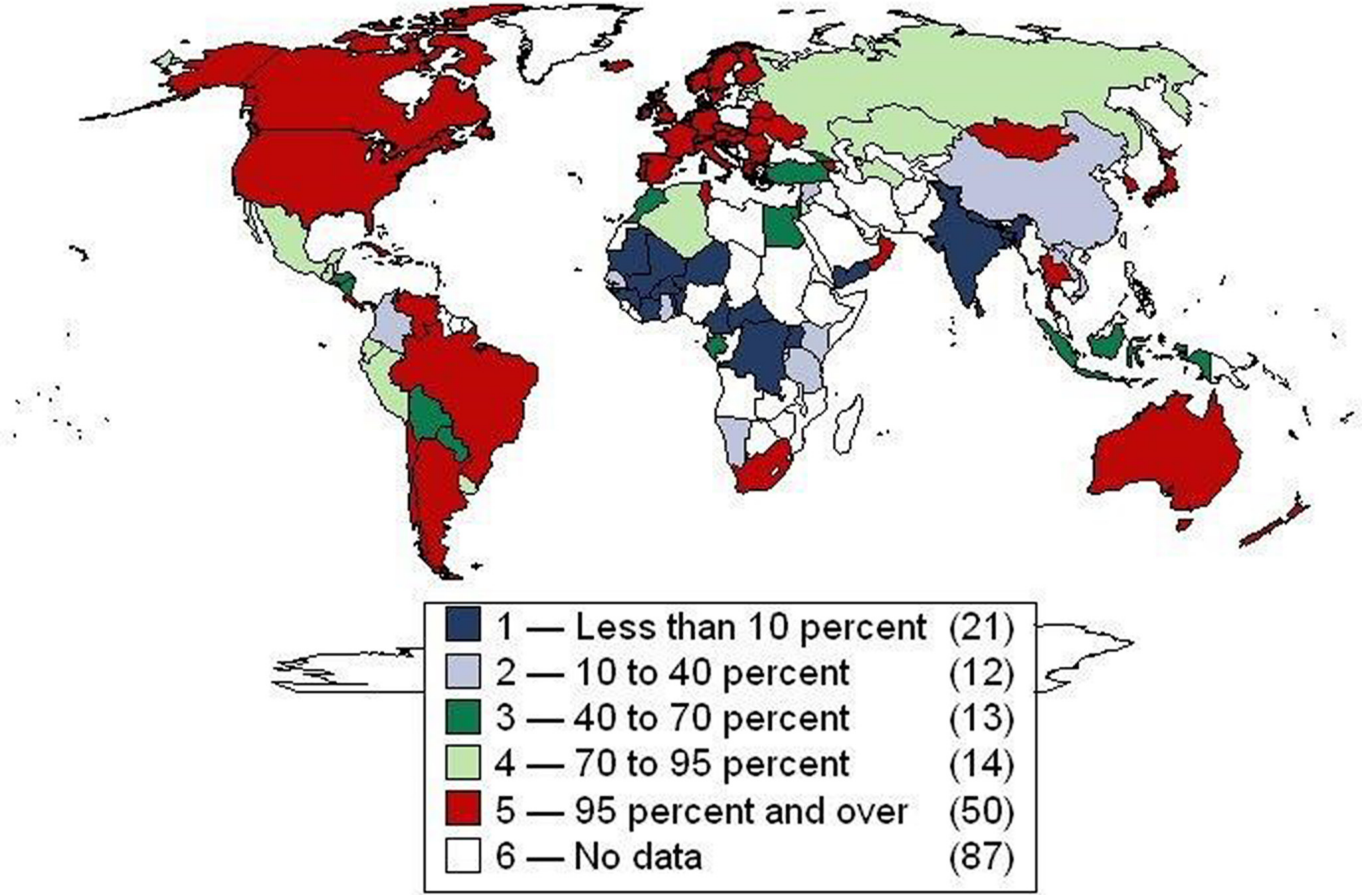
Universal Health Coverage^a^: The New Frontier for Global Health ^a^ The graph assesses the proportion of the population affiliated with national health insurance or social, private, or micro- insurance schemes. Source: This graph was first published in Garret (2009)^39^ and later updated in the International Labour Organization (2017).^41^

UHC means 3 things: (1) access for all to (2) appropriate health services (at a minimum, health promotion and primary care, with additional services depending on local epidemiology and economics), and (3) without financial hardship (financial hardship is defined as 25% or more of total household expenditures spent on out-of-pocket health expenditures).^37^ UHC is not about donors buying health insurance but about national governments organizing health financing in equitable, prepaid risk pools.^32^ The services covered under UHC should be not only curative but also public health and preventive, like immunization, nutrition, family planning, and road safety interventions. Indeed a major challenge is to prioritize such services in the face of huge demand for expensive tertiary care for urban elites. According to the International Labour Organization, over 60 countries have achieved UHC and several more are halfway in their decades-long reforms (Figure 3).^41^ Many countries, especially in Africa, are asking for technical assistance to reorient their health sector toward UHC.

Universal health coverage means access for all to appropriate health services without financial hardship.

A global movement toward the progressive realization of UHC is unfolding.^37^^,^^42^ Following the *World Health Report 2010*,^37^ the United Nations (UN) General Assembly passed a resolution supporting UHC,^43^ which is now enshrined in the Sustainable Development Goal (SDG) targets for 2030.^44^ The G7 and the G20—the international summits where leaders from the world's advanced and emerging economies meet to discuss critical global issues—support UHC, and a UHC Alliance has been born to foster and track progress to 2030. At the December 2017 Universal Health Coverage Forum in Tokyo, Japan's Prime Minister Shinzo Abe pledged US$2.9 billion for UHC while UN Secretary-General António Guterres announced UHC will be the subject of a high-level meeting at the UN General Assembly in 2019,^45^ giving impetus to WHO's goal of expanding health coverage to an additional 1 billion people in 5 years.^37^

### Development Assistance for Health

This new world health era will be driven by DRM rather than DAH and that will affect the role played by governments, industry, and international organizations.^46^ Diluted by domestic growth, DAH today accounts for less than 20% of the total health spending even in Africa^47^ and is shrinking in most recipient countries. It is already below 1% in middle-income countries like India. Donors are graduating successful countries from external assistance,^48^ with the goal of concentrating DAH in the poorest nations by 2030.^40^ These international donors also have a window of opportunity to support the transformation of health systems toward prioritizing prevention and primary care in UHC; most DAH programs are moving from service provision to capacity building and technical assistance.^48^

The new world health era will be driven by domestic resource mobilization rather than development assistance.

### Global Public Goods

In this context, DAH should shift progressively to support global public goods^49^ like global health security, international norms, pooled procurement, and research and development (particularly on diseases of poverty). While the Ebola epidemic galvanized donors to improve global health security,^50^^–^^52^ the other areas mentioned deserve equal and sustained attention. Funding global public goods makes sense as DAH dollars in countries decline or are diluted by DRM, and given their broader benefits including to the citizens of donor countries. Led by BRICS countries (Brazil, Russia, India, China, and South Africa), these and many other “emerging” economies are also contributing valuable research and development and other innovations in South-South collaboration.^53^

Development assistance should shift progressively to support global public goods.

The MDGs helped generate increased political support and funding against child and maternal mortality, HIV/AIDS, tuberculosis, and malaria. The end result was major reductions in mortality and suffering from these conditions.^2^ However, chronic noncommunicable diseases (NCDs) are a neglected area in global health that is ripe for creative action. NCDs are now the leading cause of death worldwide,^54^ and the epidemiologic transition is proceeding rapidly in Africa.^55^ Yet investments and effective solutions have lagged.^1^^,^^56^ UHC, backed by targeted DAH, offers an opportunity to tackle NCDs with multisectoral initiatives as predicated by the SDG framework.^57^

### The Private Sector and Civil Society

During the 1970s, the private sector was nearly absent from public health circles, though it was already playing a growing role in the provision of medicines and clinical services. At the turn of the millennium, many public-private partnerships (PPPs), like Gavi or the Global Alliance for TB Drug Development, were created to address market failures in the development and supply of drugs and vaccines. The change brought energy, creativity, and progress for orphan drugs and the MDGs.

PPPs 2.0 will be less top-down and more engaged in local markets and political economy, where the private sector accounts for half of total health expenditures.^1^ Private practitioners, formal or informal, already play a prominent role in service provision.^58^ Governments will need stronger stewardship capacity to regulate mixed health systems^59^ and shape markets to ensure quality and equity.^31^ As countries move toward UHC, governments will need to prioritize public financing for primary health care and population-based prevention, besides provision of curative services.^60^

PPPs 2.0 will be less top-down and more engaged in local markets and political economy.

Civil society, uniting forces with public health officials and political leaders, dramatically changed the response to HIV/AIDS, making it a top priority at all levels and driving unprecedented growth of DAH for lifesaving interventions.^61^ Civil society organizations will continue to play a critical role even as some authoritarian governments try to close the space for their work. If anything, the moral and innovative voice of NGOs, community organizations, and other civil society actors is a public good that will further grow in importance to guide multisectoral policy for UHC and to hold politicians accountable to the citizenry.^62^

While health is only one of 17 SDGs, the principle of partnership and new approaches to multisectoral collaboration will remain key in this new era. Interdependence requires closer cooperation and common aims among relevant UN agencies, development banks, professional organizations and, yes, the private sector. WHO governing bodies are exploring more inclusive engagement of non-state actors without compromising their ethics and neutrality.^63^

### The World Health Organization

As the premier UN agency for health, WHO figures prominently in historical analyses of international health.^14^^,^^22^ With greater emphasis on domestic resources, assertive member states, and the centrality of national health systems, this new era offers an opportunity to better define the role of intergovernmental organizations such as WHO and the World Bank.^64^ That is a challenge for the recently elected WHO Director-General^65^ given the complexity the international arena accrued in the previous era^18^ and the internal organizational challenges posed by decentralized management and constrained budgets^66^ relative to expectations. Tellingly, the new Director-General is from Africa and was elected for the first time by all member states, giving him, in principle, unprecedented political capital to forge ahead with his priorities, including UHC and global health security. Unlike previous eras, this new agenda has been forged and embraced by the World Bank Group as central to human capital and economic development.^44^

To succeed in a new world health era and deliver on the SDG agenda, WHO will need to act on several fronts and focus on its comparative advantages. Firstly, while technical assistance to countries and strategic leadership may not be unique to WHO, ensuring their adequate and neutral provision to member states is key to its mission. WHO needs to be able to swiftly declare public health emergencies of international concern and help improve the world's capacity to detect and respond to pandemic threats, including adherence to international health regulations and new ideas like the Coalition for Epidemic Preparedness Innovations,^67^ the Pandemic Emergency Financing Facility,^51^ and the Global Virome Project.^68^ WHO will have a crucial role in rethinking and modernizing surveillance systems and data analytics platforms, as well as the standards, prequalification, and procurement of essential drugs and vaccines in collaboration with the private sector.

As with the Framework Convention on Tobacco Control and *The World Health Report* on UHC,^37^ WHO will be expected to provide country guidance for future-oriented health systems and policies. Together with the World Bank and development partners, WHO should advocate for increasing DAH for the poorest countries while advising better-off members states to prepare for successful graduation from DAH through hybrid mechanisms like the Global Fund and the Global Financing Facility.^34^ Finally, WHO should expand the reach and quality of its advocacy and strategic communication capacity to ensure that global guidelines are clearly understood by all relevant audiences.

Internally, WHO needs to address several challenges if it is to thrive in this new world health era. It needs to define better the roles and responsibilities of its headquarters, regional, and country offices. Instead of relying only on its staff, WHO could harness today's global brain trust of experts and centers of excellence, and it should streamline the appointment of senior staff based on high-level expertise and competence rather than on geopolitical considerations. A major challenge compromising the effectiveness of WHO and threatening its independence is its budget,^66^ which is lower than the revenues of any large hospital in New York City—and three-quarters of the WHO budget comes from voluntary contributions. Member states' decisions to cut or increase assessed contributions will be pivotal. Finally, WHO needs to work well with the World Bank and related institutions, which can play a constructive role in financing health and development.

WHO needs to address several internal challenges if it is to thrive in this new world health era.

## CONCLUSIONS

The priorities and approaches used in international health have evolved with epidemiological transitions and technological innovations. But the field has also been shaped by unprecedented economic development and a historical pendulum in the role of government in social well-being. Like the Soviet Union collapse in 1991, the Great Recession of 2008 triggered one such shift in the political economy between government and market.

Global health is moving past its stage of development assistance to a new era of country ownership and global cooperation.^69^ At the national level, the economic transition of health and growing political demands for social protection create conditions favorable for domestic resource mobilization and universal health coverage with new forms of private-sector engagement. At the global level, development assistance is refocused on fragile states, the poorest communities, and global public goods like health security, normativity, and innovation.

National health systems will be the center of gravity of a new world health era, and the evolving developments discussed in this article will call for adjustments in the fluid architecture of international actors and the relations within and between nation-states. This new era brings opportunities (e.g., UHC) and challenges (e.g., growing inequalities) and new ways of financing health. Leaders at all levels should understand and capitalize on this historical moment and avoid political miscalculations like those that undermined the visionary primary health care movement 40 years ago.^15^^,^^70^

## Notes

**Peer Reviewed**

**First Published Online:** March 14, 2018

**Funding:** None.
